# Genome wide association analysis for saline-alkaline stress tolerance during the soybean germination stage

**DOI:** 10.3389/fpls.2026.1827987

**Published:** 2026-05-08

**Authors:** Ming Yuan, Xuelian Han, Haoyue Sun, Fengfeng Xu, Dongwei Han, Yazhou Zhang, Chengzhi Jiao, Wencheng Lu

**Affiliations:** 1 Qiqihar Branch of Heilongjiang Academy of Agricultural Science, Qiqihar, China; 2 State Key Laboratory of Wheat Improvement, Peking University Institute of Advanced Agricultural Sciences, Shandong Laboratory of Advanced Agricultural Sciences, Weifang, China; 3 Smartgenomics Technology Institute, Qingdao, China; 4 Heihe Branch of Heilongjiang Academy of Agricultural Sciences, Heihe, China

**Keywords:** genome-wide association analysis (GWAS), germination stage, haplotype analysis, saline-alkaline tolerance, soybean, haplotype

## Abstract

Soybean (*Glycine max*) is a globally important staple oilseed crop, yet its yield is severely limited by saline-alkaline stress. Developing soybean cultivars with enhanced saline-alkaline tolerance is therefore crucial for sustainable agriculture. To identify candidate genes associated with saline-alkaline stress tolerance in soybean, this study evaluated the saline-alkaline tolerance of a core panel of 198 soybean accessions at the germination stage, focusing on four key germination-related traits: germination energy (GE), germination rate (GR), radicle length (RL), and hypocotyl length (HL). Phenotypic analysis revealed significant variation in stress tolerance among the 198 accessions, with a subset exhibiting robust saline-alkaline tolerance and others showing significant sensitivity. This finding confirms substantial genetic diversity in saline-alkaline stress adaptability within the panel. Subsequently, whole-genome resequencing was performed on the panel, generating 385, 087 high-quality single nucleotide polymorphisms (SNPs). A genome-wide association study (GWAS) was conducted by integrating these SNP data with phenotypic traits under saline-alkaline stress, resulting in the identification of 428 candidate genes potentially associated with saline-alkaline tolerance. Notably, two previously reported salinity tolerance-related genes, *GmLecRlk* (*Glyma.07G005700*) and *GmERF34* (*Glyma.07G074200*), were successfully validated in this study. Additionally, the superior haplotypes of *Glyma.07G004400* and *Glyma.15G022000*—which showed significant associations with improved saline-alkaline tolerance—were characterized. Collectively, through phenotypic evaluation of 198 soybean accessions under saline-alkaline stress and genome-wide genetic dissection, this study provides a theoretical foundation and technical support for elucidating the molecular regulatory network of saline-alkaline tolerance in soybean and accelerating the breeding of saline-alkaline tolerant soybean cultivars.

## 1 Introduction

Currently, the global area of salinized soil is approximately 1.1 billion hectares, accounting for 7% of the world’s total land area (Balasubramaniam et al., 2023). Soil salinization is reducing global agricultural yield and affecting more than a quarter of the world’s crop species (Munns, 2002). In recent years, this phenomenon has become increasingly severe, particularly restricting seed germination, growth, and development of crops in arid and semi-arid regions (Butcher et al., 2016; Zhao et al., 2020). Salinization impairs the ability of crop roots to absorb.

water and nutrients, thereby damaging cells, organs, and tissues, slowing metabolic processes, inhibiting growth, and ultimately reducing crop yield and deteriorating quality (Liu et al., 2023). Long-term monocropping and the lack of stress-adapted crop varieties have severely limited agricultural development in saline-alkaline regions. The soda saline-alkaline soils in western Heilongjiang Province are typical representatives of saline-alkaline lands in northern China. Research and development achievements in saline-alkaline tolerant soybean varieties for this region can be extended to similar areas such as western Jilin Province and eastern Inner Mongolia, forming a “soybean industrial belt in northern saline-alkaline lands”.

Soybean (*Glycine max* L.) is an annual herbaceous plant belonging to the genus *Glycine* in the Fabaceae family. Originating in China (Liu et al., 2025), it has a cultivation history of over 5, 000 years (Sá et al., 2020) and serves as a crucial multi-purpose crop for grain, oil, and feed production. Soybean plays a key role in agricultural and industrial production, possessing biological advantages such as waterlogging tolerance and drought resistance (Lu et al., 2023; Wang et al., 2024). Additionally, soybean has biological nitrogen fixation capacity, which can effectively improve soil fertility (Arshad et al., 2025). In the global agricultural economy, soybean contributes 75% of total global protein consumption and 60% of oilseed production (USDA-FAS, http://www.usda.gov/). Over the long term, China’s demand for soybean has remained at a high level, but limited by land resources, the growth in soybean production has struggled to keep pace with demand growth (Wang et al., 2025b). Soybean is generally classified as a moderately salt-sensitive crop (Munns and Tester, 2008; Do et al., 2016). Screening and identifying new soybean varieties (lines) suitable for cultivation on saline-alkaline lands are significant ecological and economic significance for soybean saline-alkaline tolerance breeding, expanding the effective soybean planting area, and increasing total soybean yield.

Numerous functional genes and transcription factors associated with saline-alkaline tolerance have been reported in crops. For instance, in maize, *ZmNSA1* encodes an EF-hand domain-containing Ca^2+^-binding protein; loss-of-function mutants exhibit reduced shoot Na^+^content and enhanced saline-alkaline tolerance, whereas overexpression lines accumulate shoot Na^+^ and show salt sensitivity (Cao et al., 2020). In sorghum, *AT1* (homologous to rice *GS3*) encodes a heterotrimeric G-protein γ subunit, which improved saline-alkaline tolerance by regulating hydrogen peroxide (H_2_O_2)_ efflux (Zhang et al., 2023a). In rice, *SAT1* (encoding a cyclase family protein), significantly influences salinity tolerance by regulating reactive oxygen species (ROS) scavenging pathways; *sat1* knockout lines display significantly higher survival rates and lower H_2_O_2_ levels than wild-type and overexpression lines (Cao et al., 2021). Significant progress has also been made in research on soybean saline-alkaline tolerance. Specifically, a major salinity tolerance gene, *CHX1/SALT3*, was identified on chromosome 3, which significantly reduces shoot damage under salinity stress by regulating root Na^+^/Cl^-^ efflux (Guan et al., 2014; Patil et al., 2016; Jia et al., 2025). Despite the identification of numerous saline-alkaline tolerance-associated genetic loci, the underlying molecular mechanisms of plant saline-alkaline tolerance remain incompletely elucidated.

In this study, whole-genome resequencing was performed on 198 soybean accessions. A GWAS was conducted using phenotypic data obtained under treatment with an 80 mM mixed saline-alkaline solution to identify genes significantly associated with saline-alkaline tolerance. The findings of this study provide important references for future soybean saline-alkaline tolerance research and the breeding of new saline-alkaline tolerant soybean varieties.

## 2 Materials and methods

### 2.1 Plant materials

The association mapping panel used in this study consisted of 198 diverse soybean germplasm accessions, all provided by the Qiqihar Branch of Heilongjiang Academy of Agricultural Sciences. All accessions were grown at the research base in Fularji District, Qiqihar City, Heilongjiang Province, under conventional field management. After seed maturation and harvesting, plump, disease- and pest-free seeds were selected for subsequent germination experiments.

### 2.2 Saline-alkaline stress treatment

Uniform-sized, plump, and mechanically intact soybean seeds were selected as experimental materials, 60 seeds per biological replicate were used. The seed pretreatment procedure was as follows: seeds were first surface-sterilized by soaking in 75% (v/v) ethanol for 1 min, followed by five rinses with sterile deionized water, and then blotted dry on sterile filter paper to remove surface moisture (Begum et al., 2022). Pretreated seeds were evenly placed in sterile Petri dishes (9 cm in diameter) lined with a single layer of sterile filter paper serving as the germination substrate, and an additional layer of sterile filter paper was covered the seeds. In this germination system, seed orientation had no significant effect on germination performance, and seeds were placed randomly without strict orientation control during sowing. For the saline-alkaline stress treatment group, 5 mL of 80 mM mixed saline-alkaline solution (molar ratio of NaCl: Na_2_CO_3_:NaHCO_3_:Na_2_SO_4_ = 1:1:9:9, pH = 9.0) was added to each Petri dish, whereas the control group received an equal volume of sterile deionized water.

All Petri dishes were incubated in a constant-temperature growth chamber under controlled conditions: day/night temperature cycle of 25°C/16°C, light/dark period of 16 h/8 h, and relative humidity of 50%. During the stress treatment period, the sterile filter paper in each Petri dish was replaced with a new sterile one of the same specification every 2 days. To maintain stable water potential, 3 mL of the aforementioned mixed saline-alkaline solution was added to the stress treatment group, and an equal volume of sterile deionized water was added to the control group simultaneously (Li et al., 2022; Ma et al., 2025; Wang et al., 2025a). Three biological replicates were established for each treatment group and control group.

### 2.3 Phenotypic data collection and statistical analysis

Seed germination was defined as the first day when the RL reached or exceeded half the seed length (Saleem et al., 2019), and this day was also recorded as the start of saline-alkaline stress treatment. On the 3rd day of germination, the GE of each soybean accession was calculated. On the 7th day of germination, the GR, RL, and HL were measured for each accession.

### 2.4 Calculation of GE and GR

GE is expressed as a percentage and calculated using the formula: GE (%) = (N_3_/N) × 100, where N_3_ is the number of germinated seeds on the 3rd day, and N represents the total number of seeds per replicate.

GR is expressed as a percentage and calculated using the formula: GR (%) = (N7/N) × 100, where N7 is the number of germinated seeds on the 7th day, and N represents the total number of seeds per replicate.

### 2.5 Calculation of saline-alkaline tolerance coefficient

The STC, expressed as a percentage, is calculated using the formula: STC (%) = (AvgT/AvgC) × 100, where AvgT = average measured value of the saline-alkaline stress treatment group (derived from three biological replicates); AvgC = average measured value of the control group (treated with sterile deionized water, derived from three biological replicates).

### 2.6 Calculation of relative saline-alkaline damage rate

The RSD, expressed as a percentage, is calculated using the formula: RSD (%) = [(C - S)/C] × 100, where C denotes the average GR of the control group, S represents the average GR of soybean seeds under saline-alkaline stress treatment.

### 2.7 Measurement of RL and HL

RL and HL of each germinated seed were measured directly using a graduated ruler (precision: 0.1 cm). For each soybean accession, all germinated seeds from both the saline-alkaline stress treatment group and the control group were measured, and the average values for each trait (RL and HL) were computed across three biological replicates.

### 2.8 Comprehensive evaluation of soybean saline-alkaline tolerance

The STC and RSD of GE, GR, HL, and RL were calculated for each germplasm. Principal component analysis (PCA) was performed to compute the comprehensive index value of saline-alkaline tolerance, and the fuzzy mathematics subordination function method was applied to calculate the comprehensive evaluation value (D value) of saline-alkaline tolerance, thereby classifying the saline-alkaline tolerance types of the accessions.

The STC values were standardized using the fuzzy mathematics subordination function method, and the measured data were transformed with the fuzzy mathematics membership degree formula (Han et al., 2025). The formula for calculating the membership function value is as follows: $U(Xj)=\frac{X_{j}-X_{\text{min}}}{X_{\text{max}}-X_{\text{min}}}$

where j = 1, 2,…, n; Xj represents the j-th comprehensive index; is the minimum value among the n comprehensive indices; is the maximum value among the n comprehensive indices.

The weight of each comprehensive index was calculated as: $W_{j}=\frac{P_{j}}{\sum_{j=1}^{n}P_{j}}$

where j = 1, 2,…, n; Wj denotes the importance degree of the j-th comprehensive index among all comprehensive indices; Pj represents the contribution rate of the j-th comprehensive index.

The D-value, which indicates the comprehensive saline-alkaline tolerance evaluation value of each soybean variety, was derived from the comprehensive index evaluation using the formula: $\;D=\sum_{j=1}^{n}[U(X_{j})\times W_{j}]$

where j = 1, 2,…, n; U(Xj) denotes the membership function value of the j-th comprehensive index for each variety; jrepresents the weight of the j-th comprehensive index. Microsoft Excel 2019 software was used for the unified collation and analysis of the aforementioned soybean phenotypic data.

### 2.9 DNA extraction and whole genome resequencing

Seeds of all soybean accessions were sown in pots in the laboratory of the Qiqihar Branch, Heilongjiang Academy of Agricultural Sciences. Leaves from 15-day-old seedlings of each variety were selected for genomic DNA extraction. Total genomic DNA was isolated using the cetyltrimethylammonium bromide (CTAB) method (Uzunova et al., 1995). Genomic DNA samples of 198 soybean accessions were fragmented to 350 bp via sonication, followed by end-polishing, A-tailing, ligation of full-length Illumina sequencing adapters, and subsequent PCR amplification. Raw sequencing data were filtered to remove adapter sequences, reads containing ≥10 consecutive Ns, and low-quality reads (Q20 base percentage < 80%). The remaining high-quality paired-end reads were aligned to the soybean reference genome (Williams82.v4) using the Burrows-Wheeler Aligner (BWA) with the command “mem -t 4 -k 32 -M” (Li et al., 2009; Pei et al., 2021). After alignment, genomic variations for each accession were called in GVCF format using the Haplotype Caller module (GVCF mode) in Genome Analysis Toolkit (GATK) (McKenna et al., 2010). Raw single nucleotide polymorphism (SNP) genotype files were generated using the Haplotype Caller module, followed by filtering with the following parameters: sequencing depth ≥ 2 per individual, genotype quality ≥ 30 per individual, minor allele frequency (MAF) ≥ 0.05, and missing rate ≤ 0.2. A total of 4, 245, 420 SNP loci were retained after filtering, and the identified SNPs were further annotated using ANNOVAR software (version 2013-05-20) based on the Williams82.v4 reference genome (Wang et al., 2010).

### 2.10 Population structure analysis

Based on the p-distances of 4, 245, 420 genome-wide SNP loci, an individual-based neighbor-joining (NJ) phylogenetic tree was constructed using TreeBest software (version 1.9.2) with 1000 bootstrap replicates to assess statistical support (Vilella et al., 2009). Population genetic structure was analyzed using ADMIXTURE software (version 1.23), with the number of ancestral populations (K) set from 2 to 15, and the best K value was determined based on cross-validation (CV) error (Alexander et al., 2009). Principal component analysis (PCA) was performed using GCTA software to visualize population genetic relationships (Alexander et al., 2009): first, a genetic relationship matrix (GRM) was generated with the parameter “--make-grm”, followed by calculation of the first three principal components using the parameter “--pca3”. To estimate the linkage disequilibrium (LD) level in the soybean population, the squared correlation coefficient (r²) between pairwise SNPs was calculated using PopLDdecay software (Zhang et al., 2019) with the parameter settings: “-MaxDist 1000kb -MAF 0.05 -Miss 0.1”. The average r² value of pairwise SNPs was computed in 1 kb sliding windows, and the genome-wide mean r² value was computed across all windows.

### 2.11 Genome-wide association study

Based on soybean resequencing data and phenotypic records of GR and HL, single nucleotide polymorphism (SNP)-based genome-wide association study (SNP-GWAS) was conducted using EMMAX software (beta version) (Kang et al., 2010). Specifically, only SNPs satisfying the following filtering criteria were retained for subsequent GWAS: sequencing depth ≥ 2× per individual per locus, missing rate < 0.2, and minor allele frequency (MAF) ≥ 0.05.

To control the genome-wide Type I error rate, the significant P-value threshold was determined as *P* < 5.6×10^-^^6^, which was derived via Bonferroni multiple testing correction. Furthermore, phenotypic correlation analysis between GR and HL, along with Manhattan plots and quantile-quantile (Q-Q) plots for GWAS results, was conducted or generated using the R packages GGally (v2.2.1) and CMplot (v3.8.1), respectively. Local Manhattan plots and linkage disequilibrium (LD) heatmaps encompassing the genomic regions around peak SNPs were visualized using LDBlockShow software (v1.4).

### 2.12 Haplotype and candidate gene analysis

Haplotype identification was performed using a combined approach of LD decay analysis and sliding window scanning. Based on the average LD decay distance of 50 kb in 198 soybean accessions (Liang et al., 2025), fixed physical sliding windows were set in this study with a window size of 100 kb and a step size of 50 kb. Significant SNPs within each 100 kb window were assigned to distinct physical intervals. The SNP with the highest −log_10_(*P*) value was defined as the peak SNP, and a 100 kb confidence interval was delineated by extending 50 kb upstream and downstream of this peak SNP. Haplotype blocks were partitioned using LDBlockShow software (v1.4), with the analysis focusing on all SNPs within the 50 kb region upstream and downstream of each peak SNP. The analysis parameters were set as follows: the threshold for LD measure D′ was 0.8, and the critical value for MAF was 0.05; the 95% confidence interval method was used to determine the boundaries of haplotype blocks (Gabriel et al., 2002). Haplotype analysis was performed using the R package “geneHapR” (v1.0.1) (Zhang et al., 2023b), and the VCF file used for this analysis was consistent with that in the GWAS. Functional annotation of genes within the candidate intervals was completed by integrating information from the SoyBase database (https://www.soybase.org/) and reference genome annotation files. Candidate genes were identified via BLASTp alignment of their sequences with those in the *Arabidopsis thaliana* genome database (Liang et al., 2025).

### 2.13 Expression level prediction of candidate genes and development of kasp markers

The expression levels of candidate genes under salinity stress (100 mM NaCl) at 0 h, 1 h, 6 h, and 12 h were predicted using transcriptome data from the SoyOD database (https://bis.zju.edu.cn/soyod/) (Belamkar et al., 2014). SNP markers were converted into Kompetitive Allele-Specific PCR (KASP) markers via SNPWay (https://www.snpway.com/).

## 3 Results

### 3.1 Geographic distribution and phenotypic variation of 198 soybean accessions

**Figure 1:**
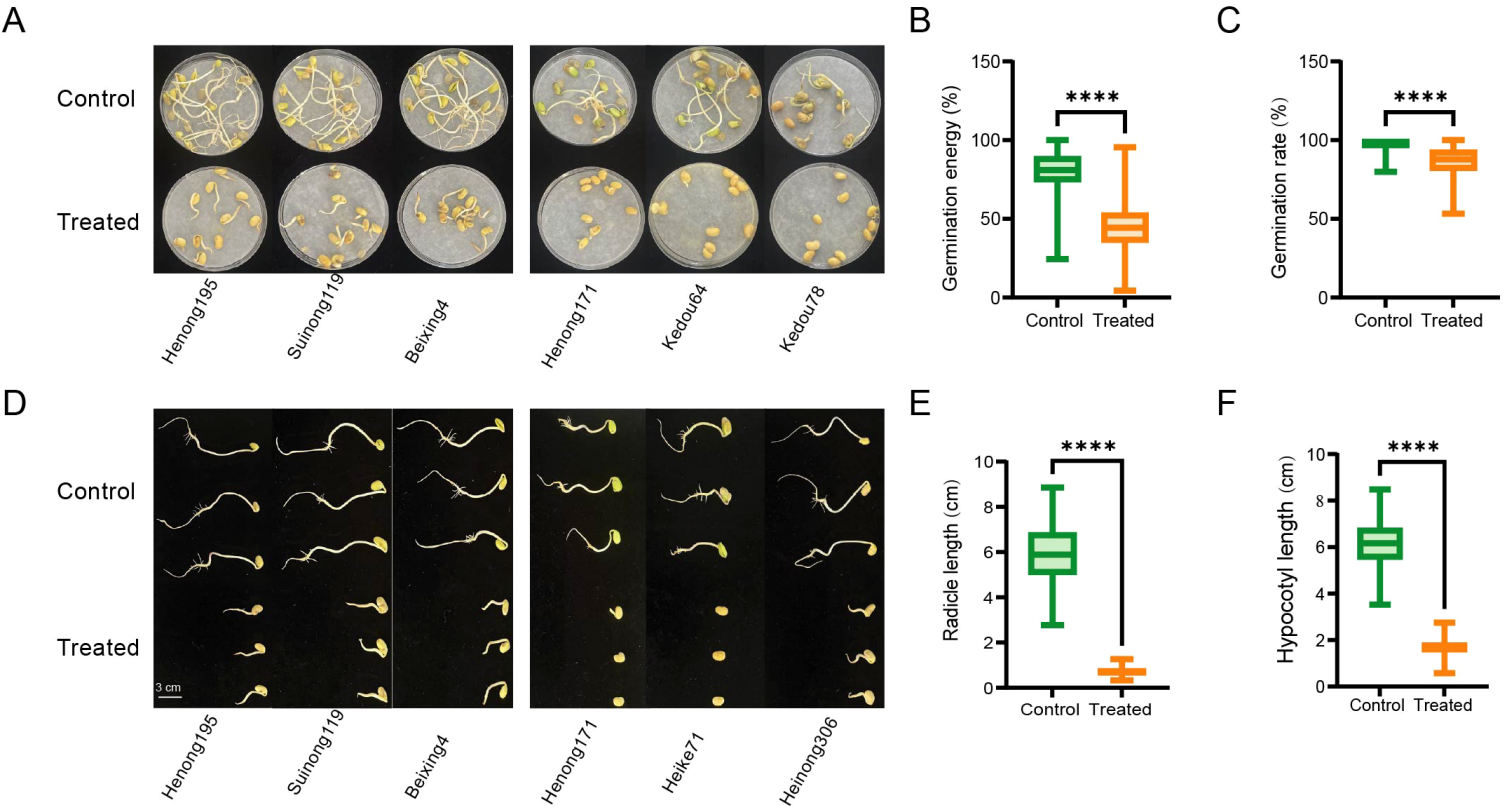
Effects of saline-alkaline stress (80 mM solution) vs. control (ultrapure water) on seed germination and seedling growth across soybean genotypes. **(A)** Germination phenotypes of saline-alkaline tolerant (e.g., Henong195, Sunong119, Baxing4) and sensitive (e.g., Henong117, Kedou64, Kedou78) soybean genotypes at 7 days post-germination (dpg). **(B-C)** Boxplots depicting differences in germination energy **(B)** and germination rate **(C)** between the control and saline-alkaline stress groups. Data are mean ± standard error (n = 3). Statistical analysis was performed via Student’s t-test; **** denotes a significant difference at *P* < 0.0001. **(D)** Hypocotyl phenotypes of the above two genotype groups at 7 dpg; scale bar = 3 cm. **(E, F)** Boxplots depicting differences in radicle length **(E)** and hypocotyl length **(F)** between the control and saline-alkaline stress groups.Data are mean ± standard error (n = 3). Statistical analysis was performed via Student’s t-test; **** denotes a significant difference at *P* < 0.0001.

A panel of 198 soybean accessions was established in this study, comprising 180 accessions from Heilongjiang Province (134 released cultivars and 46 breeding lines), 3 accessions from Jilin Province (2 released cultivars and 1 breeding line), 14 accessions from Inner Mongolia Autonomous Region (8 released cultivars and 6 breeding lines), and 1 released cultivar from Beijing. Under normal and saline-alkaline stress conditions, four key seed germination-related traits (GR, GE, RL, and HL) were recorded from the association population. Soybean germination and growth were significantly inhibited under saline-alkaline stress (**Figures 1A, D**). For the 198 soybean accessions under normal conditions, the average GE was 79.94% (**Figure 1B**), the average GR was 96.83% (**Figure 1C**), the average HL was 6.14 cm (**Figure 1E**), and the average RL was 5.92 cm (**Figure 1F**). Under saline-alkaline stress, GE, GR, RL, and HL under saline-alkaline treatment were significantly lower than those under control conditions (*P* < 0.0001). The average GE was 44.73% (**Figure 1B**), the average GR was 85.74% (**Figure 1C**), the average HL was 1.65 cm (**Figure 1E**), and the average RL was 0.71 cm (**Figure 1F**), and their phenotypic frequencies followed a normal or near-normal distribution (**Supplementary Figure 1**).

A comprehensive evaluation of saline-alkaline tolerance for the 198 soybean accessions was performed using the STC of GE, GR, HL, and RL. Based on the comprehensive evaluation results, the saline-alkaline tolerance of each accession was classified into five categories: 28 highly tolerant accessions (D-values: 0.78–0.99), 92 tolerant accessions (D-values: 0.66–0.77), 62 moderately tolerant accessions (D-values: 0.52–0.65), 11 sensitive accessions (D-values: 0.37–0.49), and 5 highly sensitive accessions (D-values: 0.16–0.31) (**Supplementary Table 1**). The saline-alkaline-tolerant accessions were mainly widely cultivated varieties in northwestern Heilongjiang Province, including high-oil varieties (e.g., Nenfeng 17, Nenfeng 18, Jiadou 30, Qinong 60), high-protein varieties (e.g., Suinong 148), and early-maturing varieties (e.g., Heike 88, Jiuyan 9). These results indicate that GE, GR, RL, and HL can effectively distinguish the responses of soybean genotypes to saline-alkaline stress.

### 3.2 Genomic variation and population genetic structure

Whole-genome resequencing was performed on 198 soybean germplasm accessions, with an average sequencing depth of 21.57×. This resulted in the identification of more than 4.2 million high-quality SNPs with MAF ≥ 0.05 and missing rate ≤ 0.2. To significantly improve the efficiency of statistical analyses, PLINK software (v1.9) was used to further filter 385, 087 high-quality independent SNPs with specific parameters (--indep-pairwise 100 5 0.2). On the genome-wide scale, the average marker density was 0.4 markers per kilobase (0.4 markers/kb). Genome-wide SNP distribution analysis revealed significant variations in the number of SNPs across 20 chromosomes (**Supplementary Table 2**). Among them, chromosome18 contained the highest number of SNPs (29, 506), while chr8 and chr11 had the lowest (13, 547 and 13, 535, respectively). This indicated heterogeneity in the genetic variation density among different chromosomes, which may be associated with the distribution of saline-alkaline tolerance-related genes. To visualize the filtered SNP distribution across each chromosome, a heatmap of SNP density was generated using the CMplot package (**Supplementary Figure 2**) (Yin, 2025). This heatmap further showed that SNP density within 100 kb windows was unevenly distributed across chromosomes, with distinct variation hotspots in some regions, suggesting potential enrichment of genetic variations associated with environmental adaptation.

**Figure 2:**
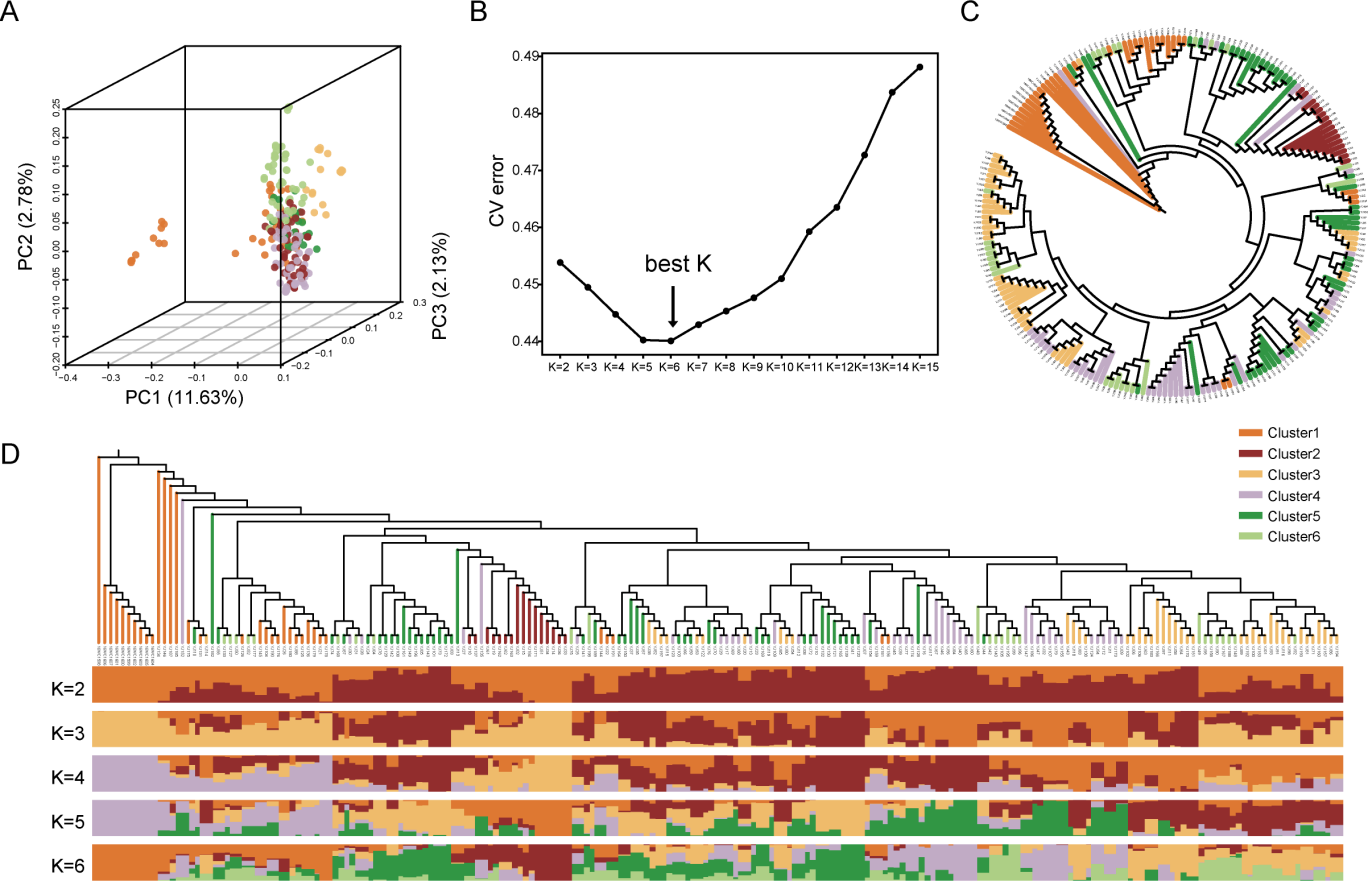
Population structure analysis of 208 soybean accessions (including 10 portions of wild soybeans). **(A)** PCA, Principal component analysis score plots: Visualizing the genetic differentiation among the accessions. **(C)** CV, Cross-validation error curve: Presenting error values used to determine the optimal number of subgroups. The X-axis represents the assumed number of groups (K), while the Y-axis denotes the CV error values (the lowest CV error corresponds to the “best K”). **(C)** NJ, Neighbor-joining phylogenetic tree: Illustrating the genetic relationships among 208 soybean accessions, with six subgroups identified based on population structure analysis. **(D)** Subgroup assignment visualization: Further delineating the six subgroups, providing a clear representation of genetic clustering patterns within the 208 soybean accessions. The first 10 samples on the leftmost side of the horizontal axis are wild soybeans.

Ten wild soybean accessions were integrated with 198 cultivated soybean germplasm accessions, yielding a total of 208 experimental materials. Principal component analysis (PCA) revealed that the first three principal components (PC1, PC2, and PC3) explained 11.63%, 2.78%, and 2.13% of the total genetic variance, respectively (**Figure 2A**). The optimal number of ancestral populations (K) was determined via cross-validation (CV) error analysis. The K value was ultimately set to 6, as this corresponded to the lowest CV error, and the 208 soybean accessions were accordingly divided into 6 distinct subgroups (**Figure 2B**). The neighbor-joining (NJ) phylogenetic tree (**Figure 2C**) was highly consistent with the population structure analysis results (**Figure 2D**), and the 198 cultivated soybean accessions evaluated in this study could be clearly assigned to these 6 subgroups: Cluster 1: Mostly late-maturing, soybean cyst nematode (SCN)-resistant, barren-tolerant, and saline-alkaline tolerant varieties from Heilongjiang Province; Cluster 2: Mostly medium-early-maturing, high-oil, and high-yield varieties from Heilongjiang Province; Cluster 3: Varieties with scattered maturity periods, mostly stable-yield and high-protein types; Cluster 4: Mostly early-maturing and stable-yield varieties from Heilongjiang Province; Cluster 5: Mostly extremely early-maturing and stable-yield varieties from Heilongjiang Province; Cluster 6: Varieties with scattered maturity periods, mostly stable-yield types.

### 3.3 GWAS for saline-alkaline tolerance-related traits

**Figure 3:**
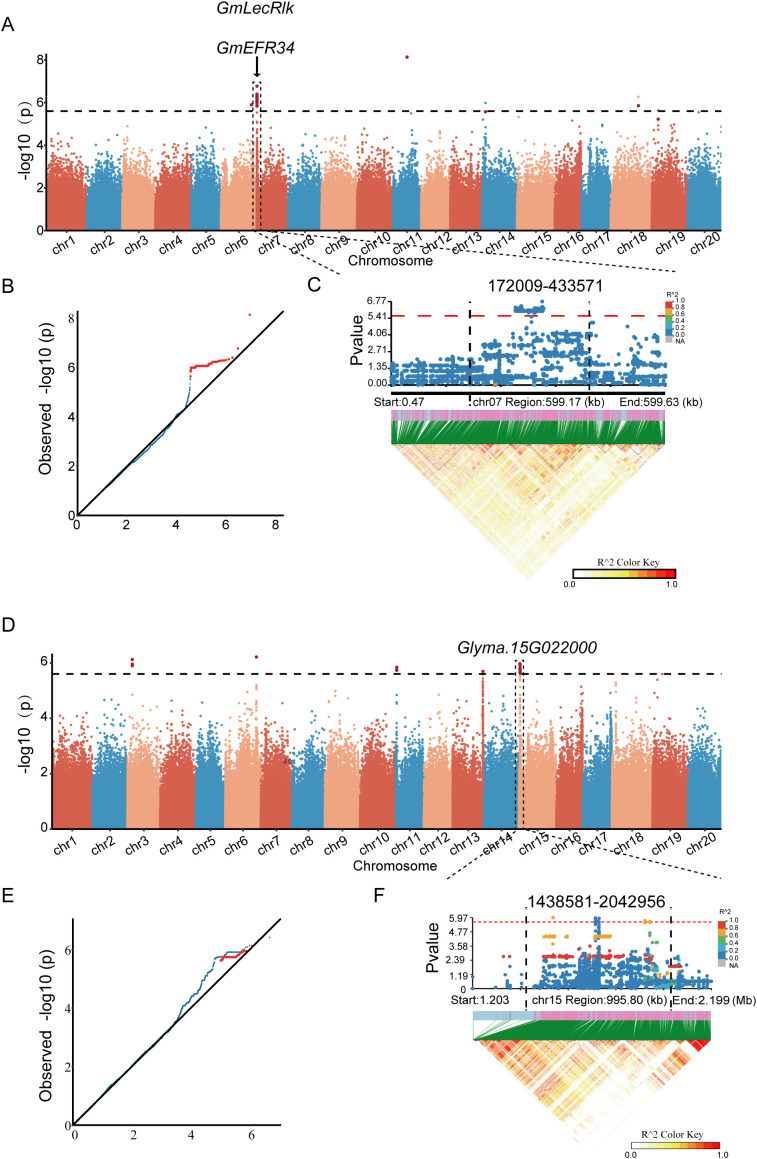
GWAS, Genome-wide association analysis results of germination rate and hypocotyl length in soybean. **(A)** Manhattan plot of GWAS for germination rate: The x-axis represents soybean chromosomes, the y-axis denotes −log10 (*P*) values, and the dashed horizontal line indicates the genome-wide significance threshold. **(B)** Quantile-quantile Q-Q plot of GWAS for germination rate: The x-axis represents expected −log10 (*P*) values, and the y-axis represents observed −log10 (*P*) values, which is used to assess the statistical validity of the association analysis (e.g., population stratification effects). **(C)** Regional Manhattan plot (top) and LD, linkage disequilibrium heatmap (bottom) surrounding the significant peak on chromosome 7. **(D)** Manhattan plot of GWAS for hypocotyl length: The x-axis represents soybean chromosomes, the y-axis denotes −log10 (*P*) values, and the dashed horizontal line indicates the genome-wide significance threshold. **(E)** Q-Q plot of GWAS for hypocotyl length: The x-axis represents expected −log10 (*P*) values, and the y-axis represents observed −log10 (*P*) values, which is used to assess the statistical validity of the association analysis (e.g., to detect population stratification). **(F)** Regional Manhattan plot (top) and LD heatmap (bottom) surrounding the significant peak on chromosome 15.

Based on 385, 087 high-confidence SNPs, a mixed linear model (MLM) was employed for GWAS of GR and HL under saline-alkaline stress. The threshold for saline-alkaline tolerance-related SNPs was set to 5.6×10–^6^ based on the genome-wide effective number of markers (GEC), leading to the identification of 40 significant SNP loci. These SNPs were primarily distributed on chr7 and chromosome 15. Manhattan plots and Q-Q plots were generated to visualize the GWAS results (**Figures 3A, B, D, E**). Clustered significant signals were identified on chromosome 7 and chromosome 15, with multiple LD blocks strongly linked to the significant loci, indicating a strong association between these genomic regions and saline-alkaline tolerance in soybean (**Figures 3C, F**). However, no significant association signals were detected for germination energy or radicle length (**Supplementary Figure 3**). Candidate intervals were defined as 100 kb upstream and downstream of each peak SNP, based on the LD decay distance. A total of 428 saline-alkaline tolerance-related genes were annotated within these intervals. Among these 428 genes, 58 candidate genes associated with seed germination under saline-alkaline stress were selected for further investigation, based on soybean genome functional annotations and functional information of homologous genes in *Arabidopsis thaliana* (**Supplementary Table 3**). Notably, two of these genes have been previously characterized to be involved in soybean salinity stress responses: *GmLecRlk* (*Glyma.07G005700*): Enhances ROS scavenging capacity in soybean. Ectopic overexpression of *GmLecRlk* in soybean hairy roots confers salinity tolerance (Zhang et al., 2022). *GmERF34* (*Glyma.07G074200*): A member of the ethylene response factor (ERF) family that participates in plant responses to salinity stress. *GmLecRlk* may improve soybean salinity tolerance by regulating *GmERF34* (Zhang et al., 2022).

### 3.4 Haplotype analysis of key candidate genes

**Figure 4:**
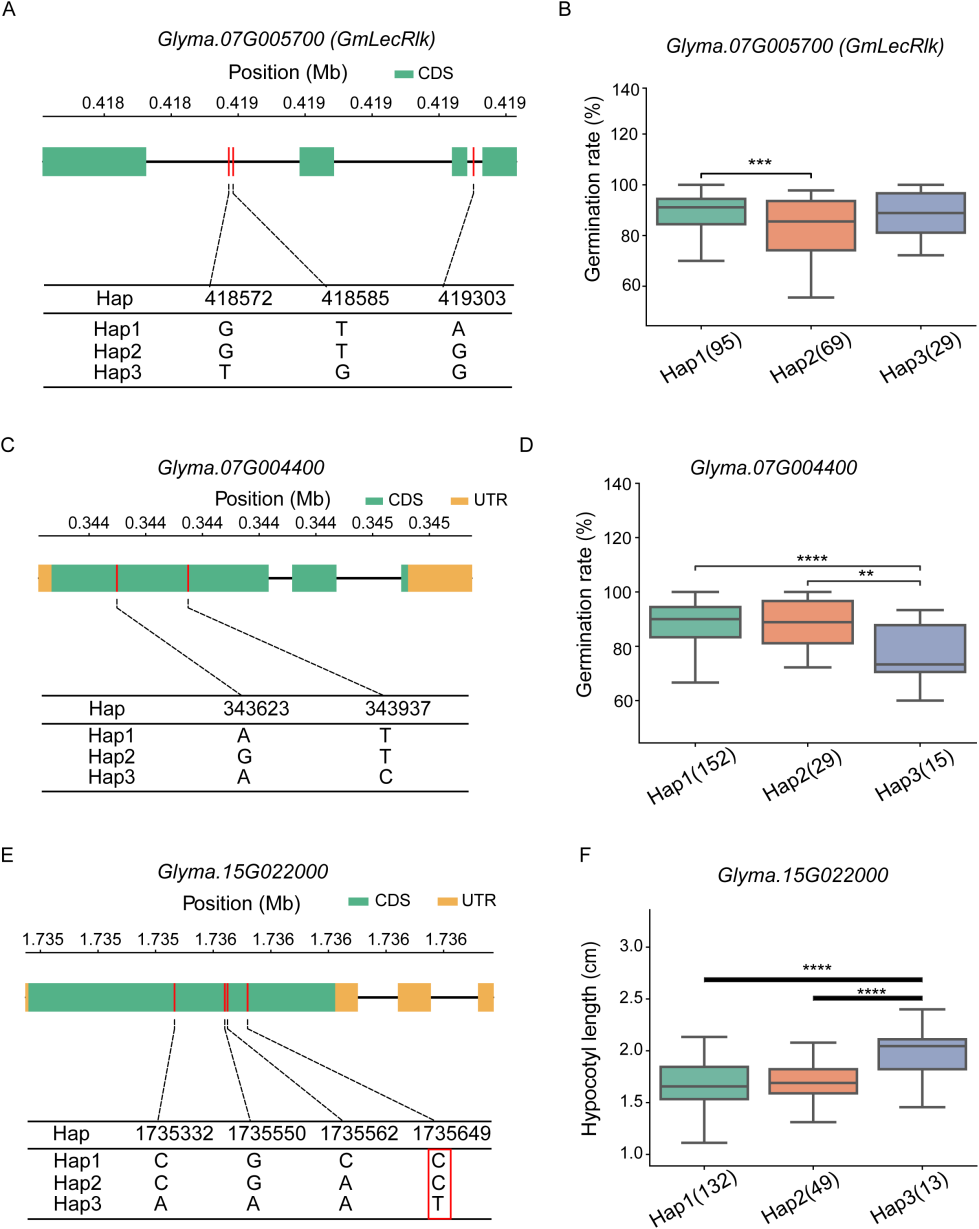
Haplotype analysis of candidate genes in soybean under saline-alkaline stress. **(A)** Haplotype analysis of the known salinity tolerance-related gene *GmLecRlk*. **(B)** Boxplots of germination rate among different haplotypes: *** indicates significant differences among haplotype groups (*P* < 0.001). **(C)** Haplotype analysis of the candidate gene *Glyma.07G004400*. **(D)** Boxplots of germination rate among different haplotypes: The symbol ** indicates statistically significant difference, P < 0.01; **** indicates extremely significant differences among haplotype groups (*P* < 0.0001). **(E)** Haplotype analysis of the candidate gene *Glyma.15G022000*: SNP, Single nucleotide polymorphism loci within the red box indicate potential functional variants, based on which KASP, Kompetitive Allele-Specific PCR markers were designed. **(F)** Boxplot of of hypocotyl length among different haplotypes: **** indicates extremely significant differences among haplotype groups (*P* < 0.0001).

The 58 candidate genes associated with seed germination under saline-alkaline stress are involved in a broad range of key biological processes, including coordinating cellular responses, regulating osmotic stress, alleviating oxidative stress, promoting ROS scavenging, and functioning as heavy metal ion transporters. Collectively, these genes play crucial roles in enhancing stress tolerance and ensuring normal plant growth and development. Subsequently, haplotype analysis was performed on these candidate genes. For the gene *Glyma.07G005700*, three major haplotypes (Hap1, Hap2, and Hap3) were identified in a population of 198 soybean accessions (**Figure 4A**). One-way analysis of variance (ANOVA) revealed that the GR exhibited significant differences between Hap1 and Hap2. Specifically, Hap1 included 95 accessions with an average GR of 89.08%, Hap2 comprised 69 accessions with an average GR of 83.68%, and Hap3 contained 29 accessions with an average GR of 88.01% (**Figure 4B**). *Glyma.07G004400*, which encodes an F-box family protein, is homologous to *Arabidopsis thaliana AT5G56420.3*. Three major haplotypes (Hap1, Hap2, and Hap3) were identified in the 198-accession population (**Figure 4C**). One-way ANOVA revealed significant differences in GR between Hap1 and Hap3, Hap2 and Hap3. Hap1 included 152 accessions with an average GR of 87.74%, Hap2 included 29 accessions with 88.01%, Hap3 included 15 accessions with 77.56%. Based on the phenotypic data of the three haplotypes and their distribution ratios in saline-alkaline tolerant vs. sensitive accessions, Hap1 (haplotype sequence: GATTC) was identified as the superior haplotype for *Glyma.07G004400* (**Figure 4D**). *Glyma.15G022000*, which encodes a bHLH87-like transcription factor, is homologous to *Arabidopsis thaliana AT3G21330.1*. Three major haplotypes (Hap1, Hap2, and Hap3) were detected in the 198-accession population (**Figure 4E**). One-way ANOVA indicated significant differences in HL between Hap1 and Hap3, Hap2 and Hap3. Hap1 included 132 accessions with an average HL of 1.67 cm, Hap2 included 49 accessions with 1.71 cm, and Hap3 included 13 accessions with 1.98 cm. Based on the phenotypic data of the three haplotypes and their distribution ratios in tolerant vs. sensitive accessions, Hap3 (haplotype sequence: GAAATA) was determined as the superior haplotype for *Glyma.15G022000* (**Figure 4F**).

### 3.5 Expression patterns of candidate genes and distribution of superior haplotypes

**Figure 5:**
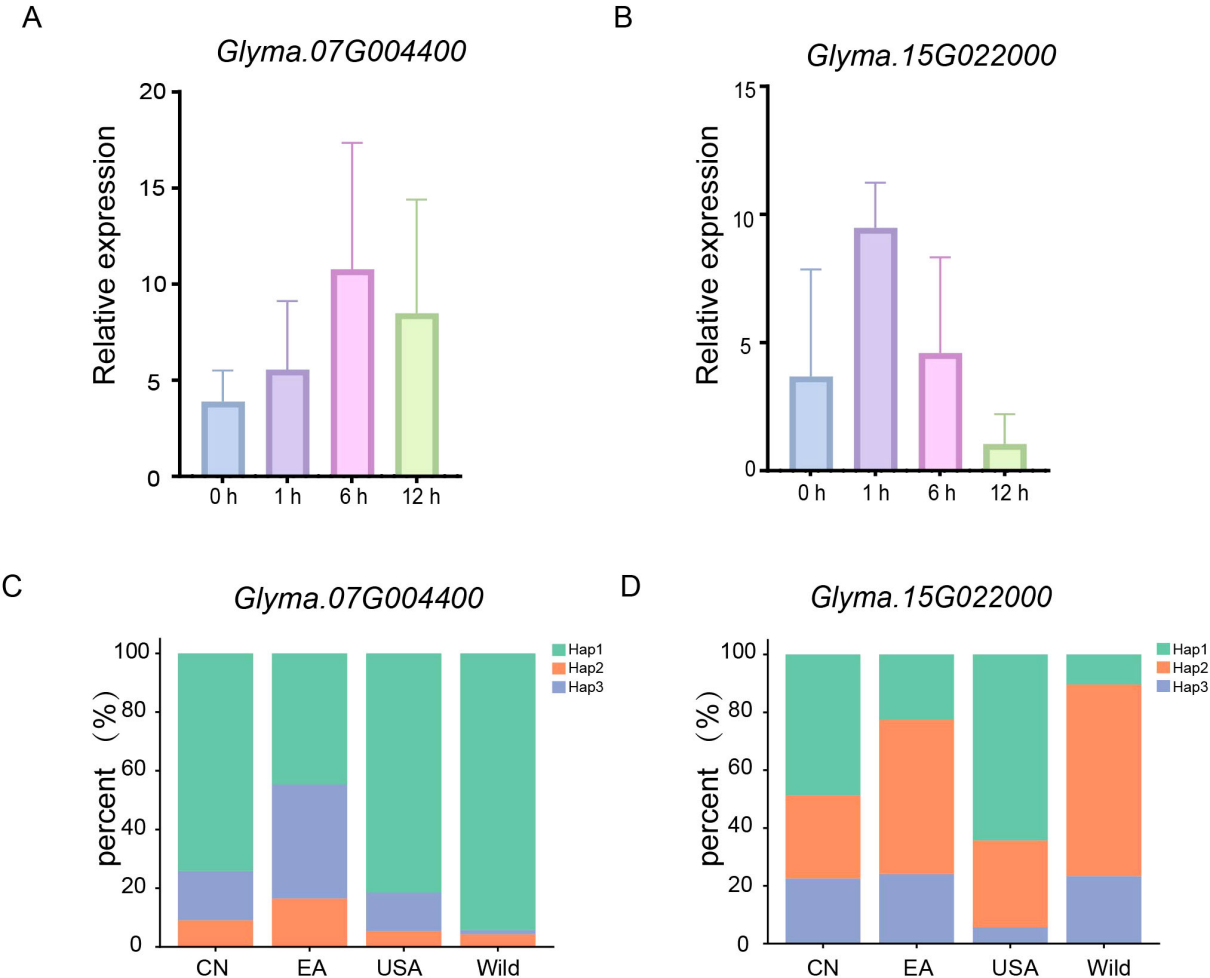
Expression patterns and haplotype distributions of two novel candidate genes (*Glyma.07G004400* and *Glyma.15G022000*) in soybean. **(A)** Expression levels of *Glyma.07G004400* in soybean root tissues under salinity stress treatments with different durations. **(B)** Expression levels of *Glyma.15G022000* in soybean root tissues under salinity stress treatments with different durations. **(C)** Stacked bar chart showing the frequency distribution of three haplotypes of *Glyma.07G004400* among soybean accessions from different geographical groups. **(D)** Stacked bar chart showing the frequency distribution of four haplotypes of *Glyma.15G022000* among soybean accessions from different geographical groups.

Based on soybean transcriptome data from the SoyOD database, *Glyma.07G004400* was significantly upregulated under salinity stress (100 mM NaCl), with its expression level peaking at 6 hours post-stress (hps) followed by a gradual decline (**Figure 5A**). In contrast, *Glyma.15G022000* reached its expression peak as early as 1 hps and then rapidly decreased (**Figure 5B**). These results demonstrate that both genes are transcriptionally regulated by salinity stress in soybean roots, exhibiting distinct expression patterns and stress-responsive dynamics. Subsequently, we analyzed the haplotype distribution of these two genes across 2003 soybean accessions. For *Glyma.07G004400*, its superior haplotype Hap1 was the most frequent in the Chinese (CN), East Asian (EA), United States (USA), and wild soybean (wild) panels (**Figure 5C**). This observation indicates that Hap1 has undergone strong artificial selection during the breeding of soybean germplasm from diverse geographical regions, reflecting its potential adaptive or agronomic value. For *Glyma.15G022000*, the superior haplotype Hap3 has undergone weak artificial selection in historical breeding practices, making it a promising haplotype with considerable application potential in soybean improvement. Notably, relatively stronger selection for Hap3 was detected in the CN panels compared to other groups (**Figure 5D**). Thus, sustained selection for Hap3 is recommended in future breeding programs to fully exploit its genetic potential. Furthermore, based on the Hap3-specific [T/C] SNP locus at position 1735649 of *Glyma.15G022000*, we developed KASP markers (**Supplementary Table 4**), which can be directly used for the rapid and efficient screening of saline-alkaline tolerant soybean germplasm resources.

## 4 Discussion

Soil salinization primarily occurs in arid and semi-arid regions of China. According to statistics, saline-alkaline soils are widely distributed across the country, including the Songnen Plain. Salinization in this plain is classified as inland soda salinization, with sodium carbonate (Na_2_CO_3_) and sodium bicarbonate (NaHCO_3_) as the dominant salts (Yang et al., 2016; Chang et al., 2022). Most areas in western Heilongjiang Province, such as Qiqihar and Daqing cities, are part of the Songnen Plain. Under saline-alkaline stress, seed germination is a critical indicator for the successful breeding of stress-tolerant cultivars (Ali et al., 2004; Zhao et al., 2014). Generally, increasing salt concentrations reduce the water potential around seeds, minimizing the water potential gradient between the seed interior and exterior, which restricts water absorption (Munns, 2002). Additionally, insufficient water uptake impairs the utilization of stored nutrients in the endosperm, inhibits respiration, restricts seed germination, and ultimately leads to reduced germination rates and delayed germination (Kazemi and Eskandari, 2011; Giménez et al., 2013).

In this study, a core panel of 198 soybean accessions was used to systematically investigate the genetic basis of saline-alkaline tolerance via GWAS. The results not only provide new insights into the molecular mechanisms underlying soybean saline-alkaline tolerance but also identify key targets for the breeding of saline-alkaline tolerant cultivars. Furthermore, the limitations of the current study are highlighted, offering important references for future research directions.

### 4.1 Validity of germination-stage phenotypic indices for saline-alkaline tolerance evaluation

From the perspective of phenotypic evaluation and population genetics, the selection of GE, GR, HL, and RL at the germination stage as evaluation indices for saline-alkaline tolerance is biologically meaningful and practically applicable. The results showed that under saline-alkaline stress, these four phenotypic traits were significantly reduced in the 198 soybean accessions compared to normal growth conditions, with significant differences between tolerant and sensitive materials. This indicates that GR and HL can effectively distinguish the response differences of soybean to saline-alkaline stress, serving as reliable phenotypic indices for saline-alkaline tolerance identification. Consistent with previous studies that used germination-related traits to evaluate stress tolerance in crops such as rapeseed and rice, these findings further validate the universality of germination-stage phenotypes in crop stress tolerance research.

### 4.2 Genomic variation and GWAS-derived candidate genes

More than 4.2 million high-quality SNPs were obtained through resequencing, and 385, 087 independent markers were retained after filtering, with an average density of 0.4 markers/kb. Notably, SNP distribution exhibited heterogeneity across different chromosomes, which may be associated with functional differentiation of soybean chromosomes. This suggests that variation hotspots on specific chromosomes (e.g., chr7, chr15) may be enriched in saline-alkaline tolerance-related genes, providing regional guidance for subsequent candidate gene fine-mapping. GWAS identified a total of 428 candidate genes, among which 58 were confirmed as core candidate genes associated with seed germination under saline-alkaline stress through functional annotation, significantly enriching the gene resource pool for soybean saline-alkaline tolerance. Importantly, the previously reported saline-alkaline tolerance genes *GmLecRlk* (*Glyma.07G005700*) and *GmERF34* (*Glyma.07G074200*) still showed significant associations with saline-alkaline tolerance in this panel. This not only verifies the reliability of previous research conclusions but also provides new interaction clues for deciphering the regulatory network of soybean saline-alkaline tolerance genes. For example, the potential regulatory relationship between *GmLecRlk* and *GmERF34* suggests that a cascade regulatory pathway may exist in soybean saline-alkaline stress response, which warrants further investigation.

### 4.3 Superior haplotypes and their breeding potential

Haplotype analysis revealed that *Glyma.07G004400* (F-box family protein) and *Glyma.15G022000* (bHLH transcription factor) possess superior haplotypes (Hap1 and Hap3, respectively) associated with enhanced saline-alkaline tolerance. The F-box family proteins are known to participate in ubiquitin-mediated protein degradation, which plays a key role in plant stress responses. The bHLH transcription factors, on the other hand, regulate the expression of stress-responsive genes by binding to specific cis-elements in their promoters. The expression pattern analysis showed that both genes are rapidly induced by salinity stress, with *Glyma.15G022000* responding within 1 hps, suggesting their involvement in the early stress response. The distribution analysis of superior haplotypes across 2003 soybean accessions indicated that *Glyma.07G004400* Hap1 has been subjected to strong artificial selection, while *Glyma.15G022000* Hap3 remains underexploited, highlighting its great potential for soybean breeding. The development of KASP markers based on *Glyma.15G022000* Hap3 provides a cost-effective and high-throughput tool for marker-assisted selection (MAS) in saline-alkaline tolerant soybean breeding, which can significantly accelerate the breeding process.

### 4.4 Limitations and future perspectives

Nevertheless, this study has several limitations that should be addressed in future research. Firstly, the stress treatment only used a mixed saline-alkaline solution of 80 mmol/L (pH=9.0), failing to simulate the complex stress environments with varying salt concentrations and pH values in nature. This may result in the non-detection of some genes specifically responsive to low or high concentrations. Secondly, phenotypic identification was only focused on the germination stage, lacking saline-alkaline tolerance phenotypic data at key growth stages such as the seedling and flowering stages, which prevents a comprehensive understanding of the genetic mechanisms underlying saline-alkaline tolerance throughout the entire soybean growth cycle. Thirdly, only 58 out of the 428 candidate genes were subjected to preliminary functional annotation. The specific functions and regulatory pathways of most genes remain unclear, and the interaction network between genes has not been established. Further in-depth exploration through transcriptome sequencing, protein-protein interaction assays, and gene editing techniques is required. Based on the above research results and limitations, future studies can be carried out in three aspects: Firstly, expand the stress treatment conditions by setting up saline-alkaline stress gradients with multiple concentrations and pH values, combined with phenotypic identification throughout the entire growth cycle, to construct a more comprehensive soybean saline-alkaline tolerance evaluation system. Secondly, conduct functional validation of key candidate genes (e.g., *Glyma.07G004400* and *Glyma.15G022000*) to elucidate their molecular mechanisms in response to saline-alkaline stress and refine the molecular regulatory network of soybean saline-alkaline tolerance. Thirdly, develop more KASP markers based on superior haplotypes and apply them in MAS to accelerate the breeding of saline-alkaline tolerant soybean varieties.

## 5 Conclusion

In this study, a core panel of 198 soybean accessions from Northeast China was subjected to whole-genome resequencing. A GWAS was performed by integrating the resequencing data with phenotypic data of GR and HL under stress from an 80 mM mixed saline-alkaline solution. A total of 40 SNPs significantly associated with saline-alkaline tolerance were identified, which were mainly distributed on chromosome 7 and chromosome 15. Additionally, 58 candidate genes related to seed germination under saline-alkaline stress were screened out, and GR and HL were confirmed to be effective indicators for distinguishing saline-alkaline tolerant soybean genotypes. The superior haplotypes of *Glyma.07G004400* (Hap1) and *Glyma.15G022000* (Hap3) were characterized, and a KASP marker based on *Glyma.15G022000* Hap3 was developed. Collectively, this study identified 58 saline-alkaline tolerance-associated genes, clarified the genomic basis of saline-alkaline tolerance in soybean at the germination stage, and provided novel insights into deciphering the molecular regulatory network underlying soybean responses to saline-alkaline stress. The research results provide clear directions for subsequent basic research and breeding practices, and are of great significance for promoting the research and application of soybean saline-alkaline tolerance.

## Data Availability

The datasets presented in this study can be found in online repositories. The names of the repository/repositories and accession number(s) can be found below: https://ngdc.cncb.ac.cn/gsa/, reference number PRJCA052275.
