# Supplementary material for: Genome wide association analysis for saline-alkaline stress tolerance during the soybean germination stage
Source: Front Plant Sci. 2026 May 8;17:1827987. doi: 10.3389/fpls.2026.1827987 (PMC13196672; doi:10.3389/fpls.2026.1827987)
Supplement: Supplementary file 1 [file DataSheet1.docx]

## Supplementary Figures


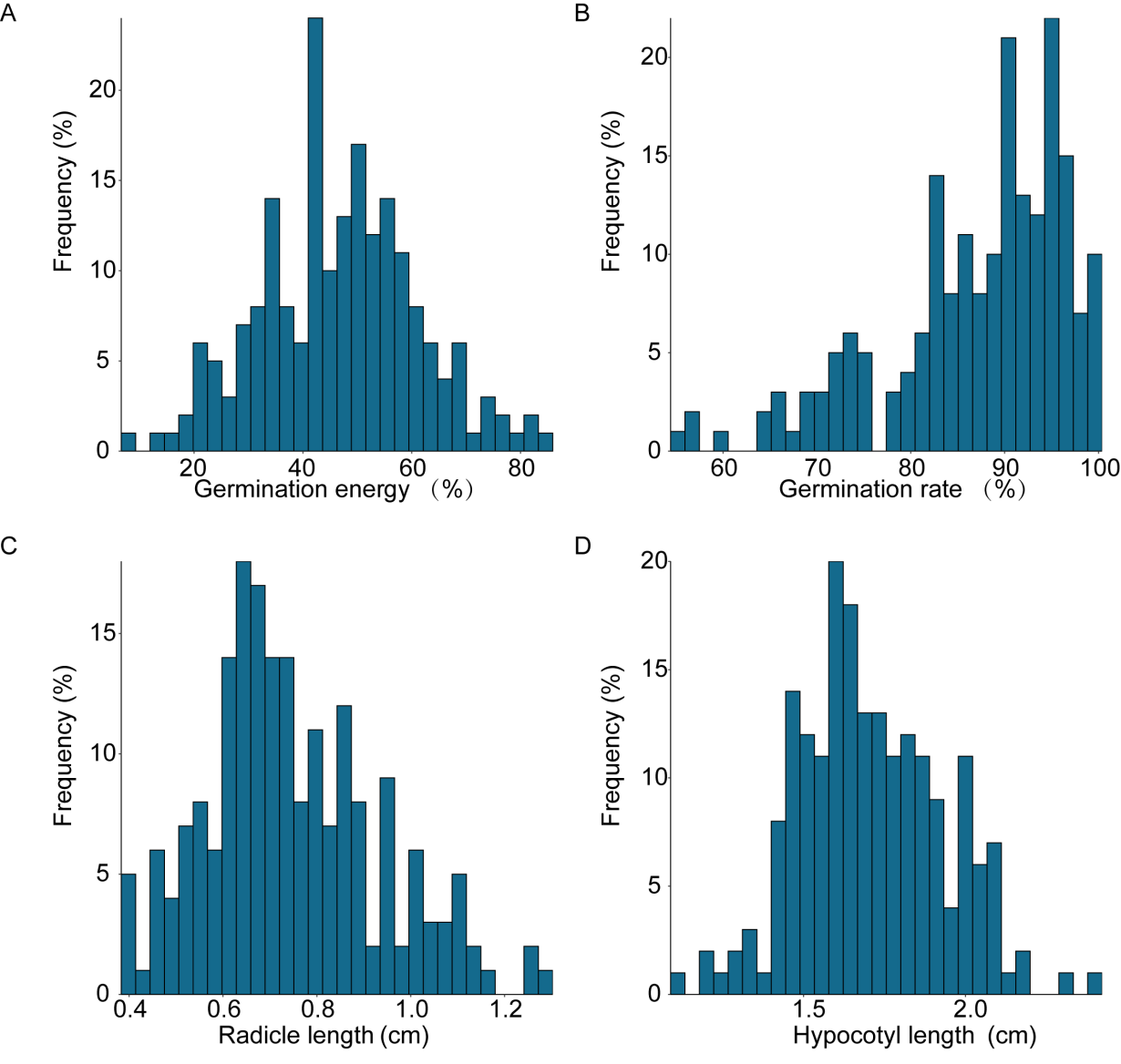


Supplementary Figure 1. Histogram of phenotypic frequency distribution for germination energy (A), germination rate (B), radicle length (C), and hypocotyl length (D) of soybeans under saline-alkali stress.


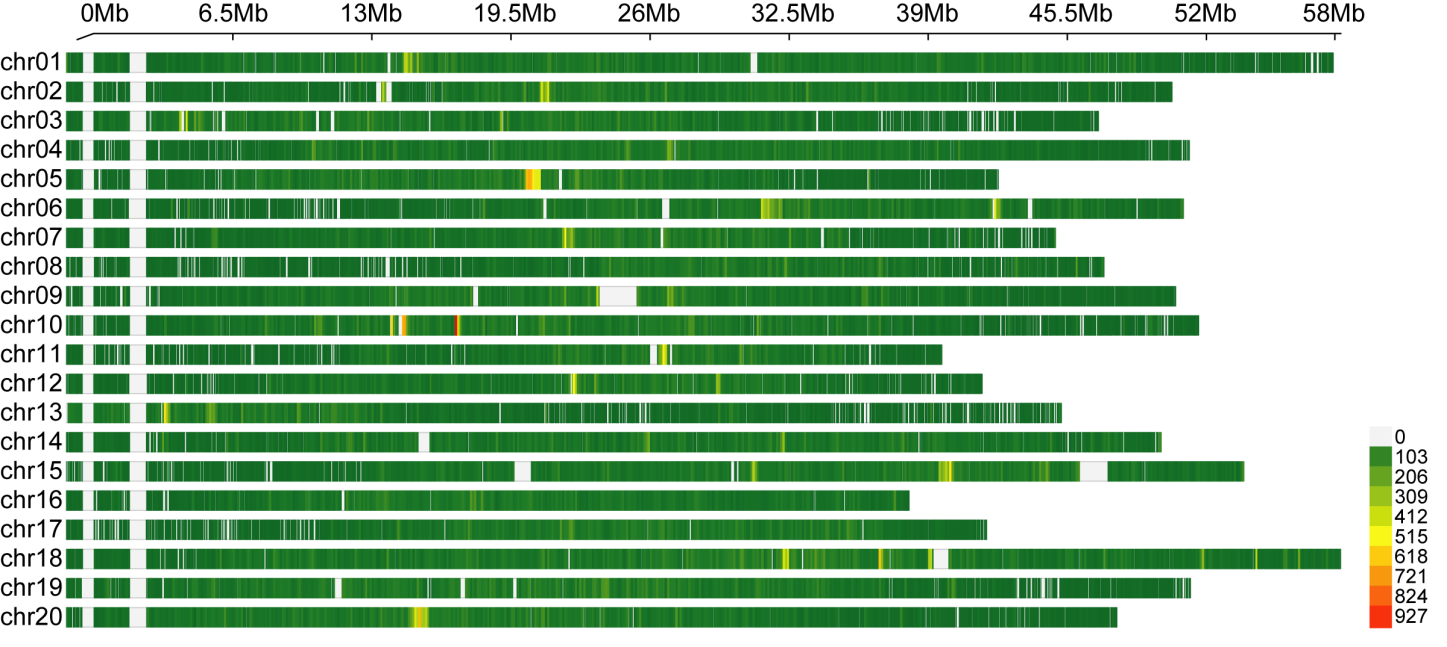


**Supplementary Figure 2.** SNP markers density for 198 soybean accessions.


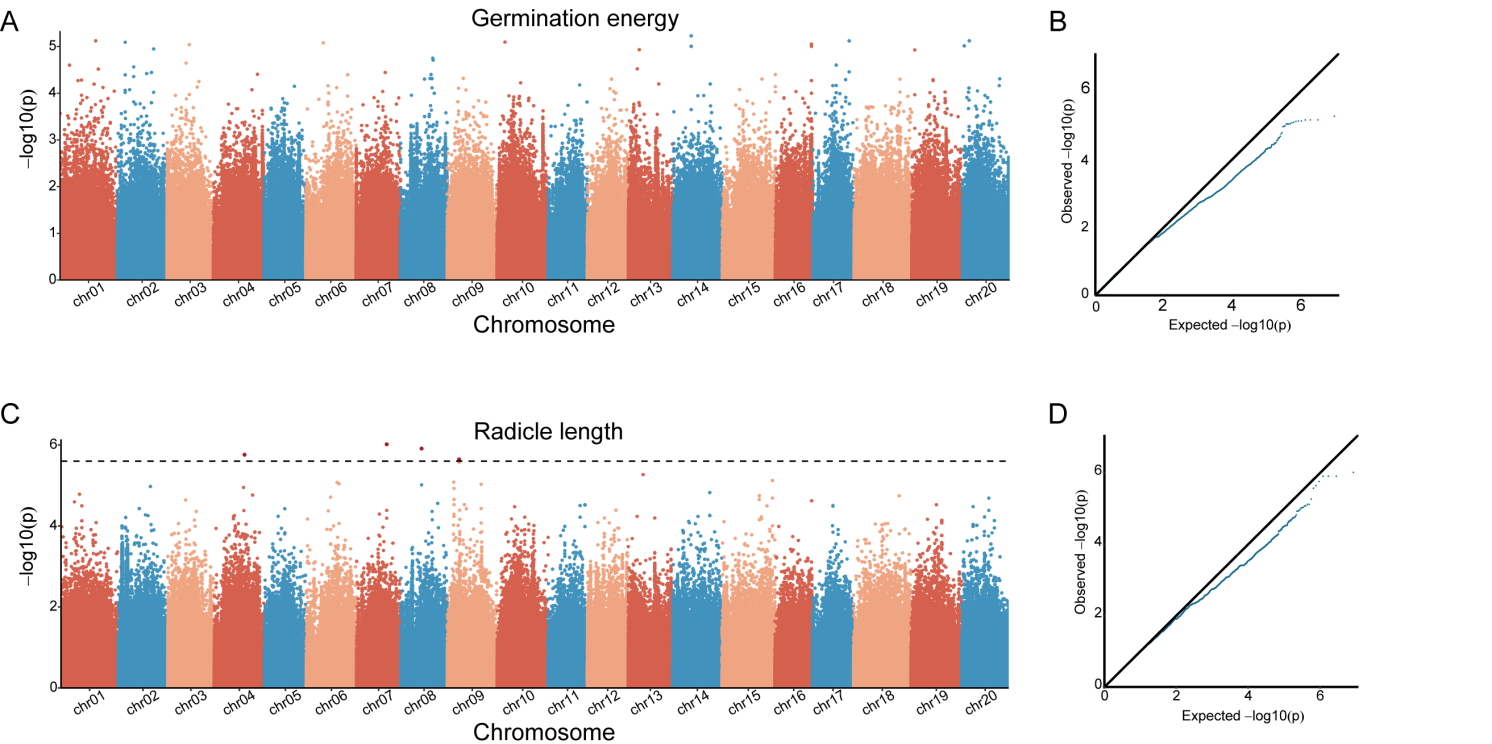


**Supplementary Figure 3.** Genome-wide association analysis (GWAS) results of germination energy and radicle length in soybean. (A) Manhattan plot of GWAS for germination energy. (B) Quantile-quantile (Q-Q) plot of GWAS for germination energy. (C) Manhattan plot of GWAS for radicle length. (D) Quantile-quantile (Q-Q) plot of GWAS for radicle length.
