# Supplementary material for: Genome wide association analysis for saline-alkaline stress tolerance during the soybean germination stage
Source: Front Plant Sci. 2026 May 8;17:1827987. doi: 10.3389/fpls.2026.1827987 (PMC13196672; doi:10.3389/fpls.2026.1827987)
Supplement: Supplementary file 2 [file Table1.docx]

**Supplementary Table 1** Sensitivity grades of 198 soybean accessions under saline-alkaline stress

| Serial Number | Germplasm Name | Comprehensive Score (D Value) | Resistance |
| --- | --- | --- | --- |
| YJ108 | Beidou 36 | 0.99 | Highly resistant |
| YJ123 | Beixing 4 | 0.89 | Highly resistant |
| YJ32 | Henong 195 | 0.88 | Highly resistant |
| YJ167 | Suinong 69 | 0.88 | Highly resistant |
| YJ143 | Jiadou 30 | 0.87 | Highly resistant |
| YJ24 | Henong 147 | 0.86 | Highly resistant |
| YJ47 | Heike 88 | 0.86 | Highly resistant |
| YJ90 | Suinong 111 | 0.86 | Highly resistant |
| YJ141 | Haojiang 5 | 0.85 | Highly resistant |
| YJ133 | Jiuyan 9 | 0.84 | Highly resistant |
| YJ68 | Kedou 53 | 0.84 | Highly resistant |
| YJ198 | Dongnong 91 | 0.83 | Highly resistant |
| YJ136 | Nenfeng 17 | 0.83 | Highly resistant |
| YJ96 | Suinong 58 | 0.82 | Highly resistant |
| YJ94 | Suinong 148 | 0.82 | Highly resistant |
| YJ04 | 919-378 | 0.82 | Highly resistant |
| YJ105 | Mengdou 48 | 0.82 | Highly resistant |
| YJ196 | Mudou 47 | 0.82 | Highly resistant |
| YJ02 | 919-372 | 0.81 | Highly resistant |
| YJ10 | Dongsheng 19 | 0.81 | Highly resistant |
| YJ140 | Kendou 60 | 0.80 | Highly resistant |
| YJ37 | Henong 81 | 0.79 | Highly resistant |
| YJ178 | Nenfeng 18 | 0.79 | Highly resistant |
| YJ110 | Longda 190 | 0.79 | Highly resistant |
| YJ48 | Heinong 304 | 0.78 | Highly resistant |
| YJ80 | Mengdou 3218 | 0.78 | Highly resistant |
| YJ165 | Longda 165 | 0.78 | Highly resistant |
| YJ119 | Qinong 60 | 0.78 | Highly resistant |
| YJ121 | Haoyu 1 | 0.77 | Resistant |
| YJ73 | Longken 310 | 0.77 | Resistant |
| YJ163 | Huinong 5 | 0.77 | Resistant |
| YJ186 | Qinong 38 | 0.77 | Resistant |
| YJ189 | Qinong 59 | 0.77 | Resistant |
| YJ142 | Heike 68 | 0.77 | Resistant |
| YJ111 | Jinyuan 71 | 0.77 | Resistant |
| YJ87 | Nongqingdou 212 | 0.77 | Resistant |
| YJ184 | Qinong 12 | 0.77 | Resistant |
| YJ185 | Qinong 25 | 0.76 | Resistant |
| YJ63 | Jinyuan 802 | 0.76 | Resistant |
| YJ85 | Nen'ao 7 | 0.76 | Resistant |
| YJ100 | Suinong 94 | 0.76 | Resistant |
| YJ22 | Qinong 7 | 0.76 | Resistant |
| YJ174 | Shengdou 87 | 0.76 | Resistant |
| YJ149 | Huinong 4 | 0.76 | Resistant |
| YJ66 | Jinyuan 601 | 0.76 | Resistant |
| YJ152 | Gandou 2 | 0.76 | Resistant |
| YJ134 | Keyan 6 | 0.76 | Resistant |
| YJ113 | Nongqingdou 214 | 0.75 | Resistant |
| YJ112 | Haojiang 8 | 0.75 | Resistant |
| YJ138 | Qinong 32 | 0.74 | Resistant |
| YJ67 | Kedou 52 | 0.74 | Resistant |
| YJ40 | Heihe 43 | 0.74 | Resistant |
| YJ151 | Jinfeng 2 | 0.74 | Resistant |
| YJ118 | Longda 137 | 0.74 | Resistant |
| YJ197 | Zhonghuang 610 | 0.74 | Resistant |
| YJ19 | Henong 134 | 0.73 | Resistant |
| YJ115 | Hefeng 55 | 0.73 | Resistant |
| YJ117 | Jiadou 16 | 0.73 | Resistant |
| YJ54 | Jiadou 10 | 0.73 | Resistant |
| YJ147 | Jiuyan 13 | 0.73 | Resistant |
| YJ59 | Jiadou 50 | 0.73 | Resistant |
| YJ150 | Henong 152 | 0.73 | Resistant |
| YJ62 | Jiadou 70 | 0.73 | Resistant |
| YJ55 | Jiadou 1 | 0.73 | Resistant |
| YJ103 | Longda 157 | 0.73 | Resistant |
| YJ182 | Qinong 3 | 0.72 | Resistant |
| YJ102 | Hedou 6 | 0.72 | Resistant |
| YJ64 | Jinyuan 603 | 0.72 | Resistant |
| YJ190 | Qinong 61 | 0.72 | Resistant |
| YJ168 | Huinong 2 | 0.72 | Resistant |
| YJ135 | Longdou 23 | 0.72 | Resistant |
| YJ144 | Qinong 49 | 0.72 | Resistant |
| YJ81 | Mengdou 33 | 0.72 | Resistant |
| YJ46 | Heike 86 | 0.71 | Resistant |
| YJ131 | Dengke 3 | 0.71 | Resistant |
| YJ41 | Heihe 45 | 0.71 | Resistant |
| YJ148 | Suinong 82 | 0.71 | Resistant |
| YJ194 | Longshengdou 19 | 0.71 | Resistant |
| YJ38 | Henong 85 | 0.70 | Resistant |
| YJ132 | Nen'ao 1 | 0.70 | Resistant |
| YJ16 | Henong 108 | 0.70 | Resistant |
| YJ15 | Henong 102 | 0.70 | Resistant |
| YJ176 | Nenfeng 16 | 0.70 | Resistant |
| YJ21 | Qinong 5 | 0.70 | Resistant |
| YJ170 | Huajiang 2 | 0.70 | Resistant |
| YJ61 | Jiadou 63 | 0.69 | Resistant |
| YJ114 | Mengdou 12 | 0.69 | Resistant |
| YJ31 | Henong 187 | 0.69 | Resistant |
| YJ58 | Jiadou 36 | 0.69 | Resistant |
| YJ127 | Heihe 49 | 0.69 | Resistant |
| YJ153 | Kendou 95 | 0.69 | Resistant |
| YJ01 | 4N1 | 0.69 | Resistant |
| YJ137 | Hefeng 51 | 0.69 | Resistant |
| YJ187 | Qinong 30 | 0.68 | Resistant |
| YJ08 | Dongpu 53 | 0.68 | Resistant |
| YJ161 | Beiyuan 5 | 0.68 | Resistant |
| YJ36 | Henong 77 | 0.68 | Resistant |
| YJ25 | Henong 165 | 0.68 | Resistant |
| YJ99 | Suinong 81 | 0.68 | Resistant |
| YJ56 | Jiadou 32 | 0.68 | Resistant |
| YJ106 | Nen'ao 6 | 0.68 | Resistant |
| YJ129 | Longda 1 | 0.68 | Resistant |
| YJ72 | Longda 185 | 0.68 | Resistant |
| YJ97 | Huajiang 71 | 0.68 | Resistant |
| YJ130 | Jiuyan 17 | 0.68 | Resistant |
| YJ82 | Mengdou 375 | 0.68 | Resistant |
| YJ180 | Nenfeng 20 | 0.68 | Resistant |
| YJ06 | Beidou 51 | 0.68 | Resistant |
| YJ26 | Henong 169 | 0.67 | Resistant |
| YJ84 | Nen'ao 5 | 0.67 | Resistant |
| YJ12 | Dongsheng 44 | 0.67 | Resistant |
| YJ34 | Henong 75 | 0.67 | Resistant |
| YJ78 | Mengdou 310 | 0.67 | Resistant |
| YJ109 | Haojiang 2 | 0.67 | Resistant |
| YJ169 | Dongpu 52 | 0.67 | Resistant |
| YJ65 | Jinyuan 503 | 0.67 | Resistant |
| YJ173 | Heinong 76 | 0.66 | Resistant |
| YJ74 | Longken 316 | 0.66 | Resistant |
| YJ183 | Qinong 10 | 0.66 | Resistant |
| YJ07 | Dongfeng 6 | 0.66 | Resistant |
| YJ193 | Kangxian 12 | 0.65 | Moderately resistant |
| YJ104 | BA04 | 0.65 | Moderately resistant |
| YJ124 | Dengke 5 | 0.65 | Moderately resistant |
| YJ17 | Henong 109 | 0.65 | Moderately resistant |
| YJ101 | Longda 130 | 0.65 | Moderately resistant |
| YJ30 | Henong 185 | 0.65 | Moderately resistant |
| YJ162 | Suiwuxingdou 3 | 0.65 | Moderately resistant |
| YJ35 | Henong 76 | 0.65 | Moderately resistant |
| YJ79 | Mengdou 315 | 0.65 | Moderately resistant |
| YJ125 | Pengdou 158 | 0.64 | Moderately resistant |
| YJ88 | Nongqingdou 24 | 0.64 | Moderately resistant |
| YJ14 | Hefeng 50 | 0.64 | Moderately resistant |
| YJ146 | Zhonghuang 35 | 0.64 | Moderately resistant |
| YJ57 | Jiadou 33 | 0.64 | Moderately resistant |
| YJ42 | Heike 104 | 0.63 | Moderately resistant |
| YJ86 | Nongqingdou 20 | 0.63 | Moderately resistant |
| YJ179 | Nenfeng 19 | 0.63 | Moderately resistant |
| YJ192 | Qinong 52 | 0.62 | Moderately resistant |
| YJ122 | Huajiang 5 | 0.62 | Moderately resistant |
| YJ126 | Nongqingdou 28 | 0.62 | Moderately resistant |
| YJ76 | Longken 3346 | 0.62 | Moderately resistant |
| YJ53 | Huajiang 89 | 0.62 | Moderately resistant |
| YJ120 | Huajiang 17 | 0.62 | Moderately resistant |
| YJ83 | Nen'ao 12 | 0.62 | Moderately resistant |
| YJ157 | Heinong 308 | 0.61 | Moderately resistant |
| YJ175 | Nenfeng 15 | 0.61 | Moderately resistant |
| YJ158 | Heinong 80 | 0.61 | Moderately resistant |
| YJ28 | Henong 173 | 0.61 | Moderately resistant |
| YJ98 | Suinong 76 | 0.61 | Moderately resistant |
| YJ18 | Henong 114 | 0.61 | Moderately resistant |
| YJ166 | Longda 3 | 0.60 | Moderately resistant |
| YJ160 | Heinong 321 | 0.60 | Moderately resistant |
| YJ75 | Longken 3301 | 0.59 | Moderately resistant |
| YJ91 | Suinong 119 | 0.59 | Moderately resistant |
| YJ11 | Dongsheng 41 | 0.59 | Moderately resistant |
| YJ172 | Jinyuan 73 | 0.59 | Moderately resistant |
| YJ51 | Heinong 87 | 0.58 | Moderately resistant |
| YJ05 | Beidou 40 | 0.58 | Moderately resistant |
| YJ39 | Hedou 1 | 0.58 | Moderately resistant |
| YJ93 | Suinong 135 | 0.58 | Moderately resistant |
| YJ33 | Henong 72 | 0.58 | Moderately resistant |
| YJ191 | Qinong 29 | 0.58 | Moderately resistant |
| YJ145 | Qinong 1 | 0.57 | Moderately resistant |
| YJ50 | Heinong 531 | 0.57 | Moderately resistant |
| YJ03 | 919-376 | 0.57 | Moderately resistant |
| YJ139 | Heihe 53 | 0.57 | Moderately resistant |
| YJ20 | Henong 142 | 0.57 | Moderately resistant |
| YJ60 | Jiadou 52 | 0.57 | Moderately resistant |
| YJ164 | Huinong 417 | 0.57 | Moderately resistant |
| YJ181 | Qinong 2 | 0.56 | Moderately resistant |
| YJ52 | Jiyu 81 | 0.56 | Moderately resistant |
| YJ128 | Heike 85 | 0.56 | Moderately resistant |
| YJ23 | Henong 144 | 0.55 | Moderately resistant |
| YJ156 | Heinong 90 | 0.55 | Moderately resistant |
| YJ09 | Dongsheng 17 | 0.55 | Moderately resistant |
| YJ188 | Suinong 26 | 0.54 | Moderately resistant |
| YJ45 | Heike 71 | 0.54 | Moderately resistant |
| YJ107 | Fengdou 3 | 0.53 | Moderately resistant |
| YJ43 | Heike 59 | 0.53 | Moderately resistant |
| YJ116 | Qinong 58 | 0.53 | Moderately resistant |
| YJ154 | Longken 3092 | 0.53 | Moderately resistant |
| YJ71 | Kendou 94 | 0.52 | Moderately resistant |
| YJ171 | Henong 100 | 0.49 | Sensitive |
| YJ77 | Mengdou 1137 | 0.48 | Sensitive |
| YJ49 | Heinong 312 | 0.43 | Sensitive |
| YJ44 | Heike 67 | 0.43 | Sensitive |
| YJ13 | Haojiang 25 | 0.42 | Sensitive |
| YJ195 | Dongnongdou 405 | 0.42 | Sensitive |
| YJ177 | Dongsheng 121 | 0.40 | Sensitive |
| YJ92 | Suinong 120 | 0.40 | Sensitive |
| YJ95 | Suinong 50 | 0.39 | Sensitive |
| YJ89 | Qing 2312 | 0.38 | Sensitive |
| YJ159 | Heinong 306 | 0.37 | Sensitive |
| YJ27 | Henong 171 | 0.31 | Highly sensitive |
| YJ155 | Wodou 5 | 0.30 | Highly sensitive |
| YJ29 | Henong 177 | 0.27 | Highly sensitive |
| YJ69 | Kedou 64 | 0.27 | Highly sensitive |
| YJ70 | Kedou 78 | 0.16 | Highly sensitive |

**Supplementary Table 2** Distribution of SNP Markers Across Chromosomes

| Chromosome | Number of SNPs |
| --- | --- |
| chr1 | 24,388 |
| chr2 | 18,770 |
| chr3 | 19,416 |
| chr4 | 19,545 |
| chr5 | 19,857 |
| chr6 | 20,180 |
| chr7 | 17,843 |
| chr8 | 13,547 |
| chr9 | 19,683 |
| chr10 | 23,410 |
| chr11 | 13,535 |
| chr12 | 15,592 |
| chr13 | 14,474 |
| chr14 | 19,971 |
| chr15 | 22,293 |
| chr16 | 14,953 |
| chr17 | 15,184 |
| chr18 | 29,506 |
| chr19 | 21,647 |
| chr20 | 21,293 |
| All | 385,087 |

**Supplementary Table 3** Information on 58 identified saline-alkaline tolerance genes

| Gene ID | Chr | peakSNP | Pvalue | Homologs | Functional annotation |  |
| --- | --- | --- | --- | --- | --- | --- |
| Glyma.07G001800 | 7 | 272009 | 6.279428772 | AT3G20240.1 | mitochondrial substrate carrier family protein B-like |  |
| Glyma.07G001900 | 7 | 272009 | 6.279428772 | AT1G78895.1 | reticulon-like protein B2 |  |
| Glyma.07G002100 | 7 | 272009 | 6.279428772 | AT2G46910.1 | Plastid-lipid associated protein PAP / fibrillin family protein; |  |
| Glyma.07G002200 | 7 | 272009 | 6.279428772 | AT5G19990.1 | 26S protease regulatory subunit 7-like |  |
| Glyma.07G002600 | 7 | 272009 | 6.279428772 | AT1G16860.1 | uncharacterized membrane protein At1g16860-like isoform X3 |  |
| Glyma.07G002700 | 7 | 272009 | 6.279428772 | AT4G27990.1 | YGGT family protein |  |
| Glyma.07G002800 | 7 | 272009 | 6.279428772 | AT1G79650.2 | Rad23 UV excision repair protein family; IPR004806 (UV excision repair protein Rad23 |  |
| Glyma.07G003200 | 7 | 272009 | 6.279428772 | AT1G51660.1 | Protein kinase superfamily protein |  |
| Glyma.07G003300 | 7 | 272009 | 6.279428772 | AT1G73050.1 | Glucose-methanol-choline (GMC) oxidoreductase family protein |  |
| Glyma.07G003400 | 7 | 272009 | 6.279428772 | AT1G17745.1 | D-3-phosphoglycerate dehydrogenase; IPR006236 (D-3-phosphoglycerate dehydrogenase |  |
| Glyma.07G003600 | 7 | 272009 | 6.279428772 | AT5G49830.1 | exocyst complex component 84B |  |
| Glyma.07G003800 | 7 | 272009 | 6.279428772 | AT4G31590.1 | probable xyloglucan glycosyltransferase 5-like |  |
| Glyma.07G004000 | 7 | 272009 | 6.279428772 | AT5G03540.1 | Exocyst complex component EXO70A1 |  |
| Glyma.07G004100 | 7 | 272009 | 6.279428772 | AT4G31480.1 | Coatomer subunit beta-1 |  |
| Glyma.07G004200 | 7 | 272009 | 6.279428772 | AT3G21190.1 | Gdp-Fucose protein O-fucosyltransferase |  |
| Glyma.07G004300 | 7 | 272009 | 6.279428772 | AT3G21180.1 | calcium-transporting ATPase 8, plasma membrane-type protein |  |
| Glyma.07G004400 | 7 | 272009 | 6.279428772 | AT5G56420.3 | F-box family protein |  |
| Glyma.07G004600 | 7 | 272009 | 6.279428772 | AT3G24550.1 | Protein kinase superfamily protein |  |
| Glyma.07G004700 | 7 | 272009 | 6.279428772 | AT3G24550.1 | Protein kinase superfamily protein |  |
| Glyma.07G004900 | 7 | 272009 | 6.279428772 | AT1G15250.2 | Zinc-binding ribosomal protein family protein |  |
| Glyma.07G005200 | 7 | 272009 | 6.279428772 | AT3G16630.2 | ATP binding microtubule motor family protein isoform 1 |  |
| Glyma.07G005300 | 7 | 272009 | 6.279428772 | AT1G79900.1 | mitochondrial substrate carrier family protein B-like |  |
| Glyma.07G005700 | 7 | 272009 | 6.279428772 | AT1G11350.1 | receptor kinase 3 |  |
| Glyma.15G018900 | 15 | 1538581 | 5.965836541 | AT3G16310.1 | nucleoporin NUP53-like isoform X2 |  |
| Glyma.15G019200 | 15 | 1538581 | 5.965836541 | AT1G59870.1 | ATP-binding ABC transporter |  |
| Glyma.15G019300 | 15 | 1538581 | 5.965836541 | AT1G79750.1 | NAD-dependent malic enzyme 1 |  |
| Glyma.15G019500 | 15 | 1538581 | 5.965836541 | AT3G16360.2 | histidine phosphotransfer protein 6 |  |
| Glyma.15G019600 | 15 | 1538581 | 5.965836541 | AT5G48120.3 | MMS19 nucleotide excision repair protein homolog isoform X1 |  |
| Glyma.15G019700 | 15 | 1538581 | 5.965836541 | AT3G21360.1 | taurine catabolism dioxygenase TauD/TfdA |  |
| Glyma.15G019800 | 15 | 1538581 | 5.965836541 | AT1G51940.1 | LysM domain receptor-like kinase 3 |  |
| Glyma.15G019900 | 15 | 1538581 | 5.965836541 | AT5G65820.1 | Pentatricopeptide repeat (PPR) superfamily protein |  |
| Glyma.15G020300 | 15 | 1538581 | 5.965836541 | AT3G16500.1 | Auxin-responsive protein n=3 Tax=Citrus RepID=V4SMI9_9ROSI |  |
| Glyma.15G020900 | 15 | 1538581 | 5.965836541 | AT4G26270.1 | ATP-dependent 6-phosphofructokinase 3 |  |
| Glyma.15G021100 | 15 | 1538581 | 5.965836541 | AT3G25690.1 | protein CHUP1, chloroplastic-like isoform X1 |  |
| Glyma.15G021300 | 15 | 1538581 | 5.965836541 | AT2G19210 | Putative leucine-rich repeat receptor-like protein kinase |  |
| Glyma.15G021400 | 15 | 1715351 | 5.907199303 | AT2G19210 | Putative leucine-rich repeat receptor-like protein kinase |  |
| Glyma.15G021600 | 15 | 1715351 | 5.907199303 | AT1G05700 | Probable LRR receptor-like serine/threonine-protein kinase |  |
| Glyma.15G021700 | 15 | 1715351 | 5.907199303 | AT2G19210 | Putative leucine-rich repeat receptor-like protein kinase |  |
| Glyma.15G021800 | 15 | 1715351 | 5.907199303 | AT1G51890 | Probable LRR receptor-like protein kinase |  |
| Glyma.15G021900 | 15 | 1715351 | 5.907199303 | AT2G19210 | Putative leucine-rich repeat receptor-like protein kinase |  |
| Glyma.15G022000 | 15 | 1715351 | 5.907199303 | AT3G21330.1 | transcription factor bHLH87-like |  |
| Glyma.15G022300 | 15 | 1715351 | 5.907199303 | AT1G51760.1 | IAA-amino acid hydrolase ILR1-like 4-like |  |
| Glyma.15G022500 | 15 | 1715351 | 5.907199303 | AT5G22720.1 | F-box/RNI-like superfamily protein |  |
| Glyma.15G022600 | 15 | 1715351 | 5.907199303 | AT1G73030.1 | charged multivesicular body protein |  |
| Glyma.15G022700 | 15 | 1715351 | 5.907199303 | AT4G29410.2 | 60S ribosomal L28-like protein |  |
| Glyma.15G022800 | 15 | 1715351 | 5.907199303 | AT1G51700.1 | dof zinc finger protein DOF3.1-like |  |
| Glyma.15G022900 | 15 | 1715351 | 5.907199303 | AT1G17720.2 | Serine/threonine-protein phosphatase 2A 55 kDa regulatory subunit B n=39 Tax=rosids RepID=I1M5D7_SOYBN |  |
| Glyma.15G023000 | 15 | 1715351 | 5.907199303 | AT1G17710.1 | 2,3-diketo-5-methylthio-1-phosphopentane phosphatase; |  |
| Glyma.15G023100 | 15 | 1715351 | 5.907199303 | AT1G17710.1 | 2,3-diketo-5-methylthio-1-phosphopentane phosphatase; |  |
| Glyma.15G023400 | 15 | 1921676 | 5.731549174 | AT1G55190.1 | PRA1 (Prenylated rab acceptor) family protein |  |
| Glyma.15G023500 | 15 | 1921676 | 5.731549174 | AT1G55180.1 | phospholipase D P1 |  |
| Glyma.15G023600 | 15 | 1921676 | 5.731549174 | AT1G55170.1 | myosin-like protein |  |
| Glyma.15G023800 | 15 | 1921676 | 5.731549174 | AT3G13750.1 | beta galactosidase 1 |  |
| Glyma.15G024100 | 15 | 1921676 | 5.731549174 | AT1G72960.1 | protein ROOT HAIR DEFECTIVE 3 homolog 1-like |  |
| Glyma.15G024200 | 15 | 1921676 | 5.731549174 | AT1G72970.1 | Glucose-methanol-choline (GMC) oxidoreductase family protein |  |
| Glyma.15G024300 | 15 | 1921676 | 5.731549174 | AT2G20300.1 | Protein kinase superfamily protein |  |
| Glyma.15G024400 | 15 | 1921676 | 5.731549174 | AT1G17620.1 | Late embryogenesis abundant (LEA) hydroxyproline-rich glycoprotein family; |  |
| Glyma.15G024500 | 15 | 1921676 | 5.731549174 | AT1G55110.1 | C2-H2 zinc finger protein |  |

**Supplementary Table 4** KASP primer sequences

| Primer | Sequences |
| --- | --- |
| *SNP-1735649-Allele1* | GAAGGTGACCAAGTTCATGCTCCATTGATAAGCTGTTGTTGTTGC |
| *SNP-1735649-Allele2* | GAAGGTCGGAGTCAACGGATTCCATTGATAAGCTGTTGTTGTTGT |
| *SNP-1735649-Common* | ATCCCTTTCGGTGAATATGAACCA |
